# Cardiac MRI Strain as an Early Indicator of Myocardial Dysfunction in Hypertrophic Cardiomyopathy

**DOI:** 10.3390/ijms26041407

**Published:** 2025-02-07

**Authors:** Siqin Liu, Oumaima Laghzali, Shahriar Shalikar, Mara-Camelia Rusu, Lucie Carrier, Thoralf Niendorf, Min-Chi Ku

**Affiliations:** 1 Max-Delbrück-Center for Molecular Medicine in the Helmholtz Association (MDC), Berlin Ultrahigh Field Facility (B.U.F.F.), 13125 Berlin, Germany; siqin.liu@mdc-berlin.de (S.L.); oumaima.laghzali@mdc-berlin.de (O.L.); shahriar.shalikar@mdc-berlin.de (S.S.); thoralf.niendorf@mdc-berlin.de (T.N.); 2 DZHK (German Centre for Cardiovascular Research), partner site Berlin, 10115 Berlin, Germany; 3 Charité-Universitätsmedizin Berlin, 10117 Berlin, Germany; 4 Experimental and Clinical Research Center, a Joint Cooperation Between the Charité Medical Faculty and the Max-Delbrück Center for Molecular Medicine, 13125 Berlin, Germany; 5 Technology Platform Electron Microscopy, Max-Delbrück-Center for Molecular Medicine in the Helmholtz Association (MDC), 13125 Berlin, Germany; mara-camelia.rusu@mdc-berlin.de; 6 Department of Experimental Pharmacology and Toxicology, University Medical Center Hamburg-Eppendorf, 20246 Hamburg, Germany; l.carrier@uke.de; 7 DZHK (German Centre for Cardiovascular Research), partner site Hamburg/Kiel/Lübeck, 20246 Hamburg, Germany

**Keywords:** cardiomyopathy, myocardial remodeling, cardiac dysfunction, imaging markers, contractility, strain, magnetic resonance imaging

## Abstract

Hypertrophic cardiomyopathy (HCM) is often characterized by augmented cardiac contractility, which frequently remains undetectable in its early stages. Emerging evidence suggests that hypercontractility is linked to mitochondrial defects that develop early in HCM progression. However, imaging markers for identifying these early alterations in myocardial function are lacking. We used cardiac magnetic resonance feature tracking (CMR-FT) to assess myocardial strain in a *Mybpc3*-knockin (KI) mouse model that mimicked human HCM. While homozygous (HOM) mice exhibited cardiac hypertrophy, heterozygous (HET) mice represented an early, asymptomatic stage of HCM. To explore mitochondrial contributions to hypercontractility, we evaluated mitochondrial integrity via scanning electron microscopy (SEM) and correlated these findings with strain abnormalities. Young HET female, but not male mice exhibited significant torsion abnormalities (*p* = 0.02), reduced left ventricular global longitudinal strain (LVGLS, *p* = 0.009), and impaired right ventricular global longitudinal strain (RVGLS, *p* = 0.035) compared to the controls. Strain abnormalities correlated strongly with mitochondrial morphological alterations, including changes in volume and area distribution (R > 0.7). Abnormal myocardial strain patterns, including torsion and GLS, serve as early markers of HCM and are closely associated with underlying mitochondrial dysfunction. The HET *Mybpc3*-KI HCM model provides important insights into the initial stages of HCM progression, highlighting strain abnormalities and sex-specific differences to enhance early diagnosis and therapeutic strategies.

## 1. Introduction

Hypertrophic cardiomyopathy (HCM) represents the most prevalent inherited cardiac condition, affecting an estimated 1 in 200 to 1 in 500 individuals [1]. Sex plays a role in the prevalence of some forms of the disease [2]. HCM is characterized by hypercontractility, an early pathological feature driven by excessive myosin–actin interactions within the sarcomere [3]. This hypercontractility leads to increased left ventricular (LV) pressure, triggering progressive LV hypertrophy and adverse cardiac remodeling, including cardiomyocyte enlargement and disarray, myocardial fibrosis, and small vessel disease [4]. Hypercontractility is typically assessed through in vitro or ex vivo methods, such as sarcomeric contractility and calcium handling assays, which provide detailed insights into myocardial performance at the cellular and molecular levels, albeit with considerable complexity [5,6]. Non-invasive in vivo assessment of hypercontractility in HCM is challenging, but several methods are used. Speckle tracking echocardiography, particularly the longitudinal strain rate of the interventricular septum, detects early LV contractility changes, though it may miss subtle or regional abnormalities. Cardiac MRI with feature tracking (CMR-FT) offers several advantages for assessing hypercontractility in HCM. Unlike traditional methods like echocardiography, which may miss subtle or regional changes in myocardial function, CMR-FT provides high-resolution, detailed strain analysis across multiple myocardial directions (longitudinal, circumferential, and radial) [7]. This allows for the comprehensive evaluation of myocardial deformation, capturing even early-stage abnormalities that are often undetectable by other imaging techniques. Additionally, CMR-FT can assess the heart’s twisting motion through torsion [8] and measure strain rates [9], providing valuable insights into the dynamics of myocardial contractility. These capabilities make CMR-FT an ideal tool for detecting early changes in HCM, offering a non-invasive, in vivo method to study myocardial performance and progression of the disease. Although LV myocardial wall thickening or hypertrophy remains the primary clinical manifestation of HCM [10], right ventricular (RV) dysfunction has emerged as a critical determinant of patient prognosis [11]. RV structural and functional abnormalities significantly impact outcomes in various cardiac conditions, including heart failure and acute myocardial infarction [12]. In HCM, global RV strain has been identified as an independent prognostic marker, irrespective of the presence of RV hypertrophy [13]. While the majority of research has focused on LV strain, understanding RV dysfunction is essential for a thorough assessment of cardiac function in HCM. Hence, our study seeks to assess LV and RV strain utilizing CMR-FT.

Many patients with HCM carry autosomal dominant genetic variants in sarcomeric proteins, resulting in increased and irregular interactions between myosin and actin filaments within the sarcomere [14]. HCM involves more than 450 variants across at least 13 genes encoding sarcomeric proteins [15,16], among which *MYBPC3* genetic variants are the most frequent [4]. Notably, approximately 75% of *MYBPC3* variants result in a frameshift, which is expected to lead to C-terminal truncated protein [17]. To mimic the human disease, we utilized a *Mybpc3*-targeted knock-in (KI) mouse carrying a point mutation [18] frequently associated with HCM and linked to severe phenotypes and poor prognosis in humans [17,19]. The genetic structure of this mouse model closely mirrors that of human patients. In our study, wild-type (WT) mice served as the normal control group. Compared to WT mice, homozygous (HOM) mice develop left ventricular hypertrophy, reduced fractional shortening, and interstitial fibrosis, which represent severe HCM. While HET mice do not display left ventricular hypertrophy (LVH) or systolic dysfunction, they show increased myofilament Ca^2^⁺ sensitivity, faster Ca^2^⁺ transient decay, and diastolic dysfunction, which mirror the subtler, early-stage pathophysiology seen in human carriers of *MYBPC3* mutations [20].

Cardiac mitochondria play a crucial role in sustaining myocardial function and contractility, constituting up to 30% of the cell volume in adult cardiomyocytes [21]. Recent studies in advanced HCM have reported mitochondrial abnormalities in both structure and function. These include swollen mitochondria and reduced cristae density, and functional deficits such as decreased high-energy phosphate metabolites and impaired expression of genes related to creatine kinase and ATP synthesis [5]. These mitochondrial deficiencies are believed to manifest early in the progression of HCM, potentially contributing to the pronounced hypercontractility observed in advanced disease stages [22]. The increased mechanical workload on cardiomyocytes is inadequately supported by a proportional rise in mitochondrial calcium uptake, potentially leading to an energetic imbalance [23]. Therefore, we aim to investigate how alterations in myocardial strain correlate with changes in mitochondrial morphology.

To test our first hypothesis—that strain serves as an early marker of HCM—we employed CMR-FT to assess global and regional strain in the LV and RV of a mouse model representing early-stage HCM. To address our secondary hypothesis—that changes in myocardial strain are closely associated with mitochondrial dysfunction in early HCM—we utilized scanning electron microscopy (SEM) for the ultrastructural analysis of mitochondria integrity in this model. This integrated approach aims to elucidate how strain abnormalities correlate with mitochondrial changes, thereby enhancing our understanding of HCM progression and identifying potential imaging markers for early intervention.

## 2. Results

### 2.1. Cardiac Ventricular Function and Myocardial Thickness in HCM Mouse Models

**Figure 1:**
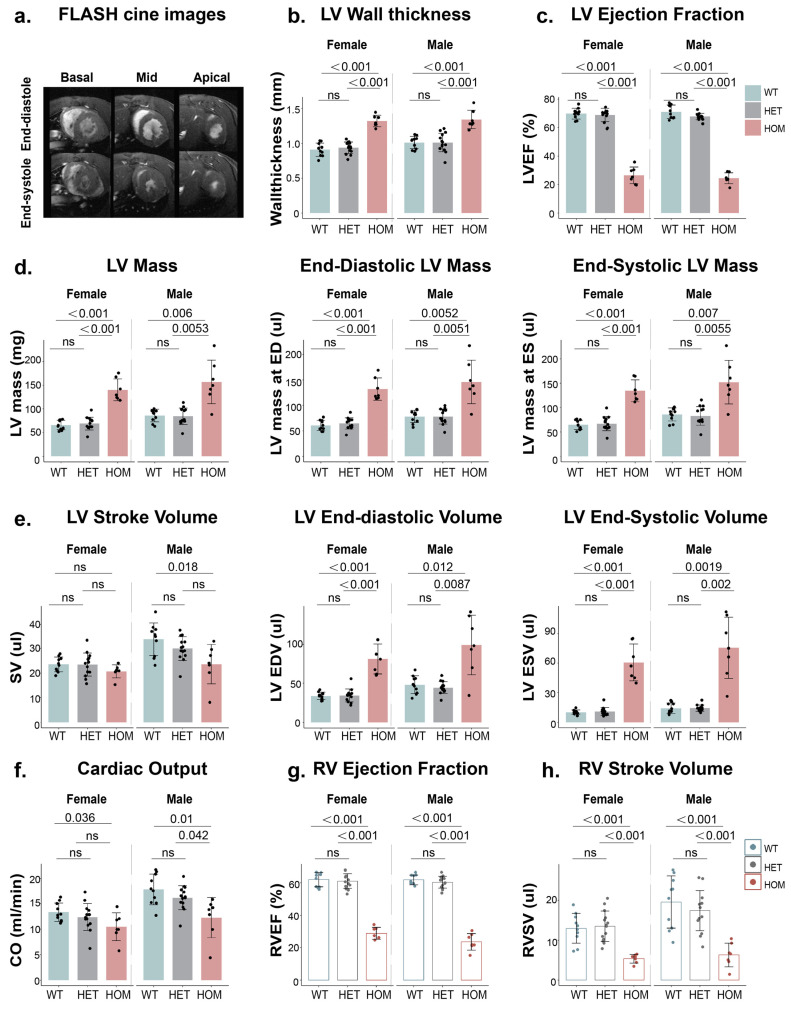
Functional analysis of ventricular performance in mice with different genotypes and genders using CMR. (**a**). Endocardial and epicardial borders were manually segmented at the end-systole and end-diastole using a stack of short-axis FLASH CINE images in both male and female mice (7–36 weeks old). (**b**). LV wall thickness is presented as a mean value with standard deviation (SD) on a slice-by-slice basis. (**c**–**h**). Functional parameters assessed include LVEF, LV mass, LV mass at ED, LV mass at ES, LVSV, LVEDV, LVESV, CO, RVEF, and RVSV. Abbreviations: LVEF: left ventricular ejection fraction; ED: end-diastolic; ES: end-systolic; LVSV: LV stroke volume; LVEDV: LV end-diastolic volume; LVESV: LV end-systolic volume; CO: cardiac output; RVEF: right ventricular ejection fraction; and RVSV: right ventricular stroke volume.

Assessing ventricular function and structure is crucial for understanding the early pathological changes in HCM. Our analysis revealed distinct differences among the HCM genotypes for LV and RV ejection fraction (LVEF and RVEF, respectively), LV end-diastolic volume (LVEDV), LV end-systolic volume (LVESV), cardiac output (CO), LV stroke volume (LVSV), RV stroke volume (RVSV), LV mass, LV end-diastolic mass (LV mass at ED), and LV end-systolic mass (LV mass at ES). HET mice carrying one mutated allele have LV and RV functions that did not differ to the WT mice. On the other hand, HOM mice that are bi-allelic for the genetic defects exhibited significantly lower LV and RV functions (Figure 1c–h). These trends were consistent across male and female mice. In males, LVEF did not differ significantly between HET and WT mice, while it was 66% lower in HOM mice than in WT mice (*p* < 0.001). In females, LVEF values were similar between HET and WT mice, while they were 60% lower in HOM than in WT mice (*p* < 0.001; Figure 1c). Similarly, RVEF did not differ between male HET and WT mice, whereas it was 62% lower in HOM than in WT male mice (*p* < 0.001). Similarly, RVEF did not differ between HET and WT female mice, whereas it was 54% lower in HOM than in WT female mice (*p* < 0.001; Figure 1g). Additionally, left ventricular wall thickness was significantly greater in HOM mice than in both WT and HET mice. LV wall thickness in HOM mice was markedly greater than in the WT group (male: *p* < 0.001, female: *p* < 0.001; Figure 1b). These findings underscore the significant functional and structural differences between WT and HOM mice, with HET mice exhibiting normal values.

### 2.2. Bi-Ventricular Myocardial Deformation in HCM Mouse Models

**Figure 2:**
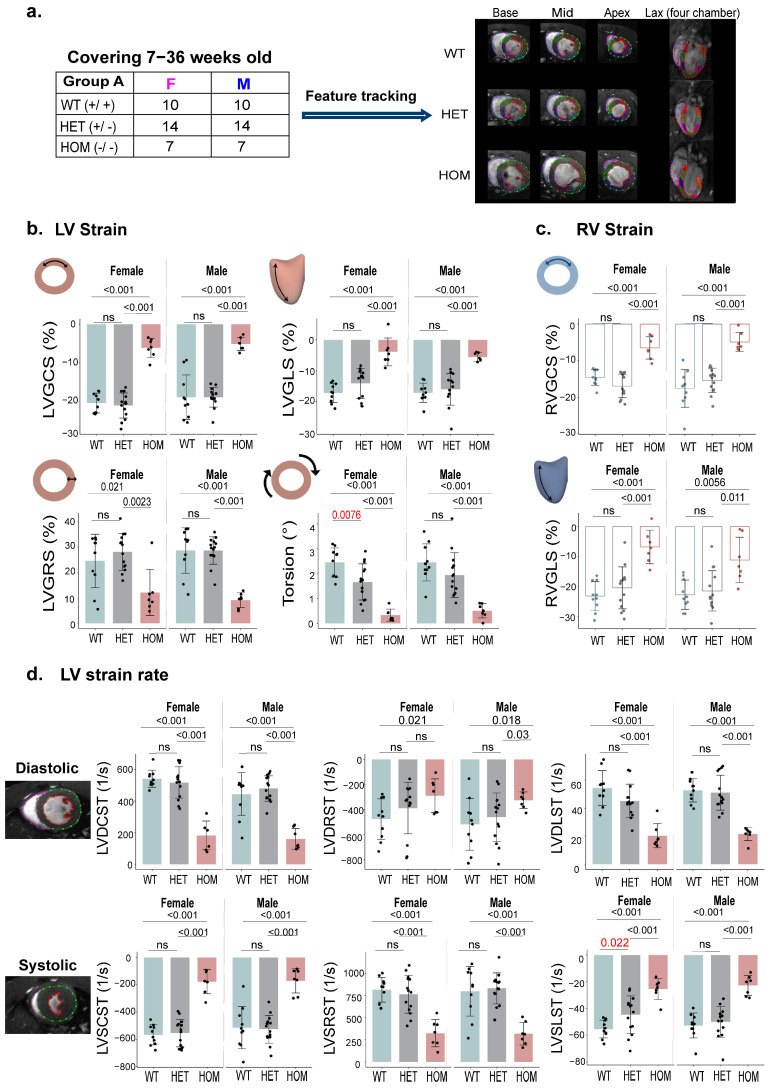

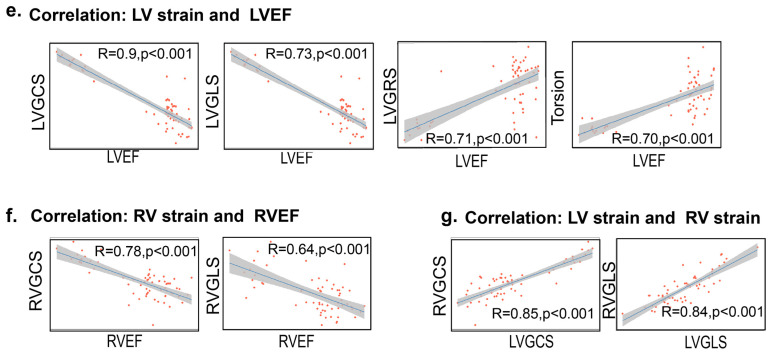
Comprehensive assessment of differences in myocardial strain in the LV and RV using CMR-FT. (**a**). CMR-FT images are shown at various slices for each genotype in both male and female mice (7–36 weeks old). (**b**). LV strain measurements, including LVGCS, LVGRS, LVGLS, and torsion, are presented as mean values with standard deviation (SD). (**c**). RV strain measurements, including RVGCS and RVGLS, are also presented as mean values with SD. (**d**). LV strain rate measurements, including LVDCST/LVSCST, LVDRST/LVSRST, and LVDLST/LVSLST are presented as mean values with SD on a slice-by-slice basis. (**e**). Correlation analysis between LV strain parameters (LVGCS, LVGRS, LVGLS, and torsion) and LVEF. (**f**). Correlation analysis between RV strain parameters (RVGCS and RVGLS) and RVEF. (**g**). Linear regression analyses of LV versus RV strain. Abbreviations: LV: left ventricular; RV: right ventricular; LVEF: LV ejection fraction; LVGCS: LV global circumferential strain; LVGRS: LV global radial strain; LVGLS: LV global longitudinal strain; RVGCS: RV global circumferential strain; RVGLS: RV global longitudinal strain; LVDCST: LV diastolic circumferential strain rate; LVSCST: LV systolic circumferential strain rate; LVDRST: LV diastolic radial strain rate; LVSRST: LV systolic radial strain rate; LVDLST: LV diastolic longitudinal strain rate; and LVSLST: LV systolic longitudinal strain rate.

To evaluate bi-ventricular myocardial deformation in HCM mouse models, we successfully utilized CMR-FT for comprehensive analysis. The tracking quality for both endocardial and epicardial borders was deemed adequate through visual inspection and manual adjustments. The age of the WT female group (n = 10) was 15.6 ± 11.5 weeks and for the WT male group (n = 10) 15.7 ± 11.2 weeks. The age of HET female group (n = 14) was 15.2 ± 10 weeks, while it was 19 ± 10.5 weeks for the HET male group (n = 14). The age of the HOM female group (n = 7) was 18.6 ± 11.1 weeks, and that of the HOM male group (n = 7) was 13 ± 8.5 weeks (Figure 2a).

Our CMR-FT analysis revealed significantly lower LV global circumferential strain (LVGCS), LV global radial strain (LVGRS), LV global longitudinal strain (LVGLS), and torsion in HOM mice than in WT and HET mice (*p* < 0.05). Similarly, RV global circumferential strain (RVGCS) and RV global longitudinal strain (RVGLS) were markedly reduced in HOM mice (*p* < 0.01). Metrics such as LV diastolic circumferential strain rate (LVDCST), LV systolic circumferential strain rate (LVSCST), LV diastolic radial strain rate (LVDRST), LV systolic radial strain rate (LVSRST), LV diastolic longitudinal strain rate (LVDLST), and LV systolic longitudinal strain rate (LVSLST) were also significantly lower in HOM mice (*p* < 0.05). Significant differences between WT and HET mice in the female group were observed for torsion (*p* = 0.0076) and LVSLST (*p* = 0.022; Figure 2b–d), suggesting their potential as early imaging markers for subtle cardiac alterations in HCM. The torsion in HET mice was marginally lower than in WT mice (*p* = 0.0076; Figure 2b), indicating early cardiac changes even in the HET genotype.

We observed strong correlations between LV strain metrics (LVGCS, LVGRS, LVGLS, torsion) and LVEF (R ≥ 0.7, *p* < 0.001; Figure 2e), as well as between RV strain metrics (RVGCS (R = 0.78), RVGLS (R = 0.64)) and RVEF (*p* < 0.001; Figure 2f). Although strain demonstrated a strong correlation with EF, it offered superior sensitivity in detecting subclinical myocardial dysfunction. While no significant differences were observed in the LV and RV functional parameters between the WT and HET groups (Figure 1), the strain parameters revealed distinct differences, highlighting their ability to detect subtle myocardial changes. Positive correlations were also found between LV and RV global strains, with RVGCS and LVGCS (R = 0.85, *p* < 0.001) and RVGLS and LVGLS (R = 0.84, *p* < 0.001; Figure 2g).

### 2.3. Myocardial Strain as an Early-Stage Marker in HCM

**Figure 3:**
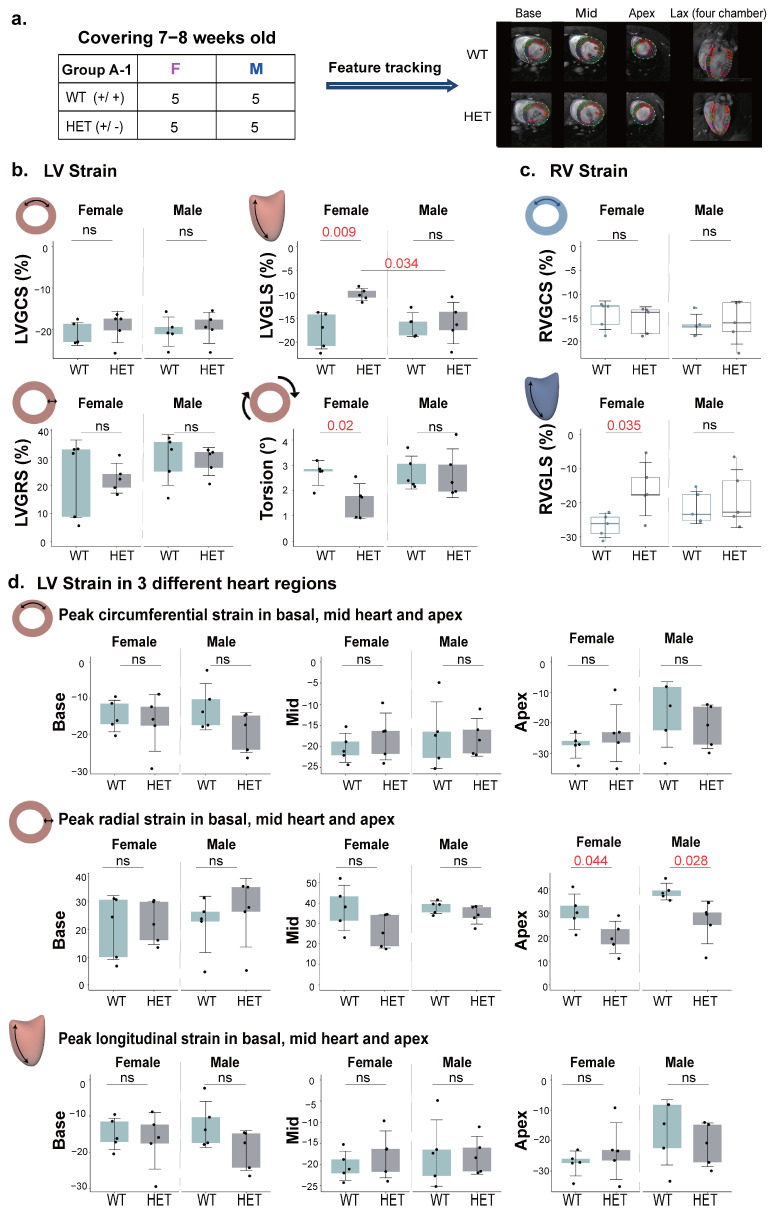
Myocardial strain analysis across different genotypes, regions, and genders. (**a**). CMR-FT images are shown at various slices for each genotype in both male and female mice (7–8 weeks old). (**b**). LV strain measurements, including LVGCS, LVGRS, LVGLS, and torsion, were conducted using the CINE images shown above. (**c**). RV strain measurements, including RVGCS and RVGLS, were also conducted using the CINE images shown above. Strain assessments for each genotype are presented as mean values with standard deviation (SD) on a slice-by-slice basis. (**d**). Strain assessments in different regions, including peak circumferential strain, peak radial strain, and peak longitudinal strain, were also evaluated. Abbreviations: LV: left ventricular; RV: right ventricular. LVGCS: LV global circumferential strain; LVGRS: LV global radial strain; LVGLS: LV global longitudinal strain; RVGCS: RV global circumferential strain; RVGLS: RV global longitudinal strain; and SD: standard deviation.

The strain differences observed between WT and HET mice may be attributed to the wide age range of the mouse models utilized in this study [24]. We therefore analyzed young mice (7–8-weeks-old) to accurately assess early-stage HCM and its initial impact on myocardial function, minimizing age-related variability and ensuring a precise evaluation of early disease markers. We focused exclusively on HET mice, which retain normal function and show no signs of HCM, to better understand early disease progression and avoid confounding effects from advanced stages. In our comprehensive analysis encompassing all strain and strain rate parameters across all age groups, significant differences were noted in torsion (*p* = 0.0076) and LVSLST (*p* = 0.022) between WT and HET mice, particularly in the female cohort. Furthermore, in the strain analysis of the 7–8-week-old HET and WT mice, significant differences were also observed in LVGLS (*p* = 0.009), RVGLS (*p* = 0.035), and torsion (*p* = 0.022; Figure 3b,c). These findings reinforced our initial observations across all age groups and underscore the potential utility of torsion and GLS as early markers for detecting the subtle cardiac alterations associated with HCM.

To gain deeper insight into the strain characteristics associated with HCM, we conducted a detailed exploration of regional strain features. Peak circumferential strain, peak radial strain, and peak longitudinal strain were segmented into basal, mid, and apical layers for analysis. Strain also offers key advantages over EF by providing regional insights into myocardial function, allowing the identification of localized wall motion abnormalities that may remain obscured in global measures like EF. Moreover, strain is less influenced by loading conditions, making it a more robust marker for nuanced myocardial performance. We compared different strain values across different genotypes within these layers. Our findings revealed a statistically significant difference in the apical layer between WT and HET mice, specifically within the peak radial strain (male: *p* = 0.028; female: *p* = 0.044) (Figure 3d). We also observed significant differences in LVGLS between females and males with HCM at an early stage of HCM (*p* = 0.034; Figure 3b).

### 2.4. Ultrastructural Myocardial Alterations in HCM Mice

**Figure 4:**
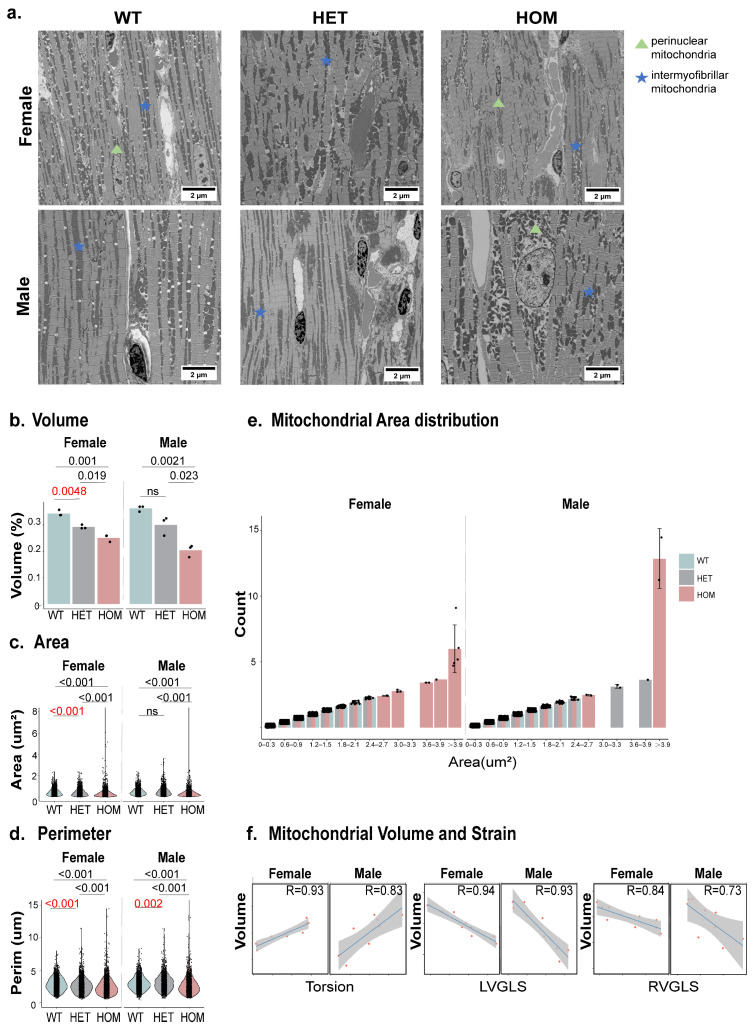
Mitochondrial morphological analysis in mice with different genotypes. (**a**). Representative electron micrographs of ventricular sections from WT, HET, and HOM mice. Scale bars: 2 µm. Key features include: 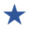 : perinuclear mitochondria; 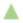 : intermyofibrillar mitochondria. (**b**). Mitochondrial volume quantified using stereology on 2D micrographs, with each dot representing the average volume from a single mouse. c. Violin plots showing the distribution of the average 2D area (**c**) and perimeter (**d**) of mitochondria for each genotype (WT, HET, and HOM), analyzed using the Kruskal–Wallis test. (**e**). Different genotypes of mitochondria grouped by regions of varying areas. (**f**). Correlation analysis between imaging markers (Torsion, LVGLS, and RVGLS) and mitochondrial volume. Abbreviations: WT: wild-type; HET: heterozygous; HOM: homozygous; LV: left ventricular; RV: right ventricular; LVGLS: LV global longitudinal strain; and RVGLS: RV global longitudinal strain.

Cardiac mitochondria play a crucial role in sustaining myocardial function and contractility, constituting up to 30% of the cell volume in adult cardiomyocytes [21]. These mitochondria are classified into intermyofibrillar (IFM) and perinuclear (PNM) types, each with distinct functions. IFM and PNM were identified in WT, HET, and HOM mice. In WT and HET mice, myofibrils were aligned with uniformly distributed IFM, with intact sarcomere structures and normal mitochondrial morphologies (Figure 4a). Collagen deposition was minimal, and areas of mitochondrial disruption were electron-lucent. However, HOM mice displayed significant disorganization of the myofibrils and mitochondria, with clustered IFM and an abnormal mitochondrial volume, shape, and area distribution. SEM also revealed substantial variability in mitochondrial area distribution in both the HET and HOM mouse groups, including an abundance of smaller mitochondria and irregularly shaped giant mitochondria (Figure 4e), particularly in females. These changes resulted in an overall reduction in mitochondrial area (Figure 4c). Both excessively large and small mitochondria exhibit functional impairments [25]. These SEM observations correlated with the myocardial strain data: the disorganized mitochondrial morphology in HOM mice aligned with the reduced myocardial strain metrics. The mitochondrial volume, area distribution, and perimeter in HET mice showed significant differences compared to WT, especially in the female group (Figure 4b–d). Torsion, LVGLS, and RVGLS were significantly correlated with mitochondrial volume (Figure 4f). These findings underscored the potential of combining myocardial strain measurements and mitochondrial morphology analysis to identify early indicators of HCM.

## 3. Discussion

This study demonstrated the use of CMR-FT to assess global and regional myocardial strain in both the left and right ventricles of a human analogue HCM mouse model. By highlighting the correlation between our strain measurements and mitochondrial morphology, we identified potential imaging markers for the early detection of myocardial alterations in HCM. This novel approach not only enhances our understanding of HCM pathology but also paves the way for the early detection of HCM.

The literature indicates that both LV strain and RV strain are more sensitive prognostic indicators in HCM patients compared to LVEF [13]. However, the sensitivity and correlation of strain in early-stage HCM patients, especially in asymptomatic individuals, remain uncertain. Our findings indicate that torsion, LVGLS, and RVGLS serve as sensitive imaging markers for early HCM detection in the mouse model, as opposed to other strain parameters. These findings align with existing research on longitudinal strain and torsion in patients with HCM [26]. Ventricular function is intricately linked to myocardial fiber structure, with myocardial tissue exhibiting non-linear and complex motions dominated by rotation and stretching throughout the cardiac cycle [15]. Specifically, longitudinal strain reflects the function of sub-endocardial myocardial fibers, circumferential strain pertains to subepicardial fibers, and radial strain encompasses the entire myocardial thickness, which relates to the thickening and thinning of the myocardial tissue [27]. The observed reduction in longitudinal function, primarily influenced by subendocardial fibers, is likely due to the increased susceptibility of this layer to perfusion abnormalities and a higher risk of interstitial fibrosis compared to other myocardial layers [28]. Additionally, the heart’s twisting motion, or torsion, helps to normalize LV wall stress by reducing the transmural gradients of fiber strain, ultimately enhancing energy efficiency. However, eccentric hypertrophy is associated with reduced torsion [29]. Consistent with prior studies, we noted that the predominant alterations in strain occur at the apical level, which can be attributed to non-uniform hypertrophy, abnormal myocardial arrangement, and increased localized stress at the apex in HCM [29]. Our study also revealed a positive correlation between RVGLS and LVGLS, as well as between RVGCS and LVGCS. These results echo previous findings that compromised LV deformation significantly impacts RV deformation. Impaired LV deformation can lead to changes in LV volume and pressure, which in turn affect RV morphology and contractility, resulting in RV diastolic dysfunction and reduced myocardial deformation [30]. CINE MRI studies indicate that LV contraction primarily involves lateral motion, while RV contraction is predominantly longitudinal [31]. The shortening and thickening of the septum further contribute to reduced RV systolic volume, potentially leading to systolic dysfunction in the RV due to increased afterload [32]. Overall, our findings underscored the intricate relationships between LV and RV mechanics in the context of HCM, highlighting the importance of early detection and intervention to manage these interdependencies effectively.

Our study revealed that mice with early HCM exhibit morphological changes in mitochondria that correlate closely with myocardial strain. Mitochondria are critical in HCM pathogenesis and have been identified as potential early markers of the disease [22]. These findings highlight the critical importance of mitochondrial integrity in understanding the molecular mechanisms underlying HCM progression and its potential as an early biomarker. The genetic mutations associated with HCM, particularly in the *Mybpc3* gene, lead to hypercontractility, myofilament Ca^2^⁺ sensitization, and Ca^2^⁺ mishandling, all of which contribute to oxidative and energetic stress as well as mitochondrial dysfunction. This altered cardiac metabolism exacerbates cardiac hypertrophy and fibrosis, further complicating calcium handling and elevating oxidative stress [33]. Our findings in HET mice align with previous studies that noted higher rates of early fast exponential relaxation in septal myectomy samples from HCM patients with MYBPC3 haploinsufficiency, reinforcing the connection between mitochondrial changes and the disease’s underlying mechanisms [22].

Our SEM observations revealed an altered mitochondrial morphology and the presence of excessively large and small mitochondria. These mitochondria might exhibit functional impairments and altered mitochondrial dynamics, reflecting disruptions in mitochondrial fission and fusion, which likely serve as adaptive responses to pathological stress. Such alterations may act as compensatory mechanisms to mitigate cellular energy deficits arising from impaired sarcomeric energy utilization [34]. In the early stages of HCM, increased oxidative stress leads to significant mitochondrial structural and functional abnormalities. Moreover, failure to properly upregulate mitochondrial quality control exacerbates these energy deficiencies [35]. We also observed increased glycogen accumulation in the cardiomyocytes of HOM mice, as previously reported in this model [36] and in patients with dilated cardiomyopathy [37]. However, further metabolic assays and specific glycogen staining are required to quantify these findings more precisely. We observed a reduced mitochondrial volume in HET mice, specifically in females but not in males, compared to WT mice, suggesting that female HET mice exhibit early mitochondrial morphological alterations in the heart. This may help to explain the myocardial contractile dysfunction observed in HET mice. Mitochondria are transported along the microtubule cytoskeleton, altering their arrangement during myofiber differentiation in mice [38]. In HCM mice and human engineered heart tissue, chronic activation of tubulin tyrosination enhances cardiac function [39]. Given the strong correlation between strain and mitochondrial morphology, this relationship may arise from the elevated mechanical workload in HOM myocytes not being adequately matched by sufficient mitochondrial Ca^2+^ uptake, leading to energetic imbalances and subsequent contractile dysfunction. Moving forward, we aim to investigate mitochondrial dynamics and microtubule organization further to elucidate their roles in these pathophysiological processes, providing deeper insights into the molecular mechanisms driving HCM.

Recent studies have highlighted significant sex-specific differences in various aspects of cardiac physiology, emphasizing the need for increased awareness in this area [40,41]. These differences can influence disease progression, responses to treatment, and outcomes in HCM, the variations of which are crucial for developing tailored approaches to diagnosis and management, ultimately improving care for both male and female patients [40]. Prior CMR research has identified sex differences in myocardial strain among both healthy subjects and in patients with diastolic dysfunction [24]. Our study revealed significant mitochondrial differences and an earlier onset of strain changes in female HCM mice. This suggests that the more severe manifestations of HCM in females may stem from a combination of altered estrogen signaling, mitochondrial dysfunction, increased fibrosis, and sex-related variations in gene expression, all contributing to greater myocardial damage and dysfunction [42]. Therefore, these sex-related variations in myocardial strain should be taken into account in future investigations to enhance our understanding and management of HCM.

### Limitations of the Study

This study offers valuable insights into the mechanics of HCM, despite its several limitations. While human HCM exhibits diverse mechanical manifestations due to complex genetic factors [43], our research is based on a controlled population of mice, allowing for more consistent data collection. This controlled setting minimizes external variability, thus enhancing the reliability of our findings regarding the association between myocardial strain and mitochondrial morphology. Although the *Mybpc3* KI model may not fully represent the broader HCM population, it provides a focused view of early-phase HCM, revealing subtle mechanical dysfunctions that may be overlooked in more heterogeneous models. Our findings highlighted the potential of torsion and GLS as early imaging markers, underscoring their relevance in understanding HCM progression. While we did not assess left atrial strain, which has been validated as a prognostic marker [44,45], our study lays the groundwork for future research to include this important measure. Additionally, we recognize that age and sex significantly impact strain measurements. This study used a consistent slice thickness across all age groups. While this ensured uniform imaging parameters, it may have affected spatial coverage along the long axis of the heart in younger animals with smaller hearts. By utilizing comprehensive CMR protocols with parametric mapping, our ongoing research will explore the evolution of CMR markers over time, enhancing our understanding of how these changes might predict disease outcomes and inform treatment strategies.

## 4. Materials and Methods

### 4.1. The Mouse Model Carries a Human HCM Genetic Variant

**Figure 5:**
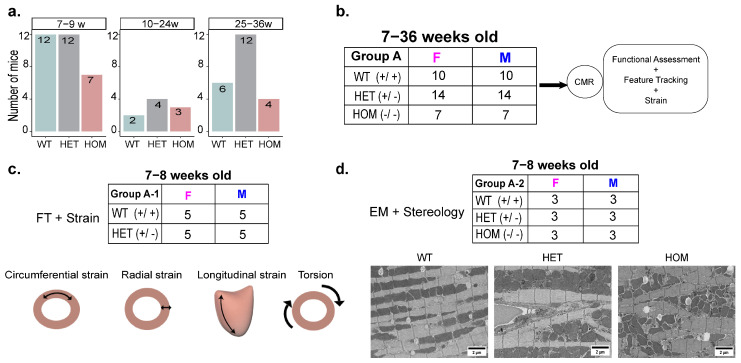
Experimental protocol. All mice underwent cardiac MRI according to the illustrated protocol. (**a**). The number of mice with different age groups across the WT, HET, and HOM groups. (**b**). Strain analysis was performed on mice aged 7 to 36 weeks old across the WT, HET, and HOM groups. (**c**). Additional strain analysis was conducted on mice aged 7 to 8 weeks old from both the WT and HET groups. Strain analysis was conducted in multiple myocardial directions, including longitudinal, circumferential, and radial. Torsion was quantified as the rotational displacement between the apex and base, normalized by the mean ventricular radius and the longitudinal axis length. (**d**). Ventricular specimens were collected from three mice in each group at 7 to 8 weeks old for electron microscopy. Abbreviations: WT, wild-type; HET, heterozygous; and HOM, homozygous.

The animal studies received approval from the Berlin State review board at the ‘Landesamt für Gesundheit und Soziales (LAGeSo; State Office for Health and Social Affairs Berlin)’. Mice were handled according to the rules and regulations set forth by the animal welfare authority and institutional rules. We utilized an HCM model with a *Mybpc3* knock-in mutation involving a single nucleotide replacement in the *Mybpc3* gene on a C57BL/6J background. In total, 62 mice (31 males and 31 females, 7–36 weeks old) underwent CINE CMR for functional and strain assessment. The selected age range of 7 to 36 weeks captures the progression from early to more advanced stages of HCM, corresponding to the transition from early- to mid-adulthood in human disease progression. This approach allowed us to observe broad trends and refine our focus. To identify early imaging markers of strain, we conducted a detailed analysis in 7-to-8-week-old mice, an age that corresponds to the early phase of the disease in humans. The experimental design is illustrated in Figure 5.

### 4.2. In Vivo Cardiac MRI

In vivo CMR was conducted using a 9.4 T small animal MR system (Biospec 94/20, Bruker Biospin, Ettlingen, Germany), utilizing a 72 mm volume resonator for transmission and a 4-channel surface cardiac radiofrequency (RF) array for signal reception. Mice were anesthetized with 3% isoflurane (CP-Pharma, Burgdorf, Germany) at a flow rate of 500 mL/min medical air and 300 mL/min oxygen, with maintenance at 1 to 1.5% isoflurane post-induction. Hemodynamic stability was rigorously maintained during CMR examinations to minimize the potential interference with imaging data. Core body temperature was regulated at 36 ± 0.5 °C through a heated water tubing system. Continuous monitoring of heart rates, respiratory rates, and core body temperature was conducted using a remote monitoring system (Model 1030, SA Instruments Inc., Stony Brook, NY, USA).

### 4.3. Assessment of Cardiac Functional Integrities

Cardiac short-axis (SAX) views covering the entire mouse heart were obtained by sequentially capturing 7–8 slices using self-gated bright-blood CINE gradient-echo imaging (IntraGate-FLASH, repetition time/echo time (TR/TE) = 8.5/1.6 ms, flip angle (FA) = 15°, receiver bandwidth = 98 kHz, FOV = 11 × 22 mm^2^, matrix size = 192 × 384, spatial resolution = 0.057 mm, slice thickness = 0.8 mm, movie frames (cardiac phases) = 16). The cardiac function assessment was conducted using Segment Version 4.0 (Medviso, Lund, Sweden) [46] and analyzed on a slice-by-slice basis. Endo- and epicardial borders were manually delineated at end-systole and end-diastole using SAX and long-axis (LAX) CINE images (four chamber view). LVEF, RVEF, LV mass, LVEDV, LVESV, CO, LVSV, RVSV, LV mass at ED, and LV mass at ES were calculated. Left ventricular wall thickness was measured at end-diastole, with the maximal wall thickness determined across all SAX views.

### 4.4. Feature Tracking and Strain Analysis

The feature tracking (FT) technique was based on a block-matching approach [7]. It first identified anatomical features along the myocardial boundaries within CINE images, established small squared regions centered around these features in an initial image, and then tracked them across the cardiac cycle by identifying the most similar image pattern in the subsequent frames [47]. Strain analysis was performed on CINE images using Segment software 4.0 (Medviso, Lund, Sweden) [48]. Standardized myocardial segmentation and nomenclature was used, including 16 segments of the basal, mid, and apical levels of the myocardium [49]. RV global strain and strain rate (SR) were assessed from the free wall, while LV global strain and SR were evaluated from the LV free wall in addition to the septum. Regional strain was assessed from the SAX and LAX views and segmented as appropriate. The software facilitated the automatic delineation of endocardial and epicardial borders during end-diastole using a point-and-click approach across the four-chamber LAX and SAX views to obtain strain values and strain rates. The global peak values of systolic strain and strain rate were determined as the highest observed values throughout systole.

### 4.5. Scanning Electron Microscopy

Following in vivo CMR, the murine hearts were extracted and fixed with 4% formaldehyde and 1.25% glutaraldehyde (Sigma-Aldrich, Taufkirchen, Germany) in 0.1 M phosphate buffer of pH 7.2 at 4 °C for 48 h. The left ventricular free wall was dissected into 1–2 mm^3^ pieces, which were processed for SEM. The tissue cubes were osmicated on ice with 1% osmium tetroxide in 0.1 M HEPES of pH 7.2, after which they were washed thoroughly with Milli Q water. Dehydration was carried out through a graded ethanol series (30%, 50%, 70%, 90%, and 100%). This was followed by a gradual infiltration with ethanol–epoxy resin Polybed812 (Polysciences) mixes (30% resin, 70% resin, and 100% resin). Infiltration with 100% resin was carried out overnight prior to polymerization at 60 °C for 48 h.

The LV free wall was sectioned using a Reichert Ultracut S ultramicrotome and an Ultra 45 diamond knife (Diatome, Nidau, Switzerland). The resulting ultrathin sections (100 nm) were collected on Type P silicon polished wafers (Science Services, München, Germany) and post-stained with 2% aqueous uranyl acetate and 3% Reynolds lead citrate (Delta Microscopies, Mauressac, France). Imaging was performed using the Helios 5 Hydra CX Dual Beam system (Thermo Scientific, Hennigsdorf, Germany). Micrographs captured at a pixel size of 4.5 nm/pixel were employed for the stereological analysis of total mitochondrial volume and for the quantification of ultrastructural mitochondrial morphological parameters.

### 4.6. Quantitative Analysis of Cardiomyocyte Mitochondrial Networks and Mitochondrial Volume

Quantification of the mitochondrial morphological parameters and mitochondrial volume was carried out using the open-source software Fiji 2.14.0 [50]. The volume of mitochondria in LV cardiomyocytes was determined from 2D SEM micrographs through point counting stereology [51]. For this analysis, three animals/condition/sexes were included and different mitochondrial populations (IFM and PNM). A uniform grid of points was projected on the micrographs using Fiji [50]. Points falling inside of mitochondria (Pm), inside the cytoplasm (Pc), and in the nucleus (Pn) were counted. After counting ≥ 700 points/animal we estimated the fraction of the cell volume that was occupied by mitochondria (Vm) according to the formula: Vm = Pm/(Pm + Pc + Pn) [52].

### 4.7. Ultrastructural Mitochondrial Morphological Parameters

Qualitative data of ultrastructural mitochondrial features were supplemented by the analysis of morphological parameters (area and perimeter). The quantification of morphological parameters was carried using Fiji [50], as described previously by Lam et al. [53].

### 4.8. Statistical Analysis

Analysis of cardiac function and strain metrics derived from CMR CINE images was conducted by observers blinded to the mouse genotype. Experimental analysis and statistical evaluations were performed using R Studio (http://www.rstudio.com/) (accessed on 4 October 2024). Group mean differences were analyzed using one-way or two-way analysis of variance (ANOVA), followed by post-hoc Tukey HSD multiple comparisons to determine significance levels, with *p* < 0.05 considered statistically significant. Variation analysis of the mitochondrial cross-sectional area was performed with Levene’s test. In the linear regression analyses, the multiple correlation coefficient (R) strength was evaluated based on its magnitude, with R ≥ 0.7 considered strong, 0.3 < R < 0.7 moderate, and R < 0.3 weak.

## 5. Conclusions

In conclusion, abnormal myocardial strain patterns, including torsion and GLS, serve as early markers of HCM. Furthermore, these strain indicators are closely linked with mitochondrial morphology, providing a foundation for future studies to enhance early diagnosis and optimize disease management strategies.

## Data Availability

Data are available from the corresponding author upon reasonable request.
